# COVID-19 pandemic stressors are associated with reported increases in frequency of drunkenness among individuals with a history of alcohol use disorder

**DOI:** 10.1038/s41398-023-02577-1

**Published:** 2023-10-06

**Authors:** Jacquelyn L. Meyers, Vivia V. McCutcheon, Kristina A. Horne-Osipenko, Lawrence R. Waters, Peter Barr, Grace Chan, David B. Chorlian, Emma C. Johnson, Sally I-Chun Kuo, John R. Kramer, Danielle M. Dick, Samuel Kuperman, Chella Kamarajan, Gayathri Pandey, Dzov Singman, Stacey Subbie-Saenz de Viteri, Jessica E. Salvatore, Laura J. Bierut, Tatiana Foroud, Alison Goate, Victor Hesselbrock, John Nurnberger, Martin H. Plaweck, Marc A. Schuckit, Arpana Agrawal, Howard J. Edenberg, Kathleen K. Bucholz, Bernice Porjesz

**Affiliations:** 1 Department of Psychiatry and Behavioral Sciences, State University of New York Downstate Medical Center, Brooklyn, NY USA; 2 Department of Psychiatry, University of Connecticut School of Medicine, Farmington, CT USA; 3 Department of Psychiatry, Washington University School of Medicine, St. Louis, MO USA; 4 Department of Psychiatry, Robert Wood Johnson Medical School, Rutgers University, Piscataway, NJ USA; 5 Department of Psychiatry, University of Iowa Carver College of Medicine, Iowa City, IA USA; 6 Departments of Genetics and Genomic Sciences, Neuroscience, and Neurology, Icahn School of Medicine at Mount Sinai, New York, NY USA; 7 Department of Biochemistry and Molecular Biology and Medical and Molecular Genetics, Indiana University School of Medicine, Indianapolis, IN USA; 8 Department of Psychiatry, Indiana University School of Medicine, Indianapolis, IN USA; 9 Department of Psychiatry, University of California San Diego Medical School, La Jolla, CA USA

**Keywords:** Human behaviour, Diseases, Predictive markers

## Abstract

Some sources report increases in alcohol use have been observed since the start of the COVID-19 pandemic, particularly among women. Cross-sectional studies suggest that specific COVID-19-related stressful experiences (e.g., social disconnection) may be driving such increases in the general population. Few studies have explored these topics among individuals with a history of Alcohol Use Disorders (AUD), an especially vulnerable population. Drawing on recent data collected by the *Collaborative Study on the Genetics of Alcoholism* (COGA; COVID-19 study *N* = 1651, 62% women, age range: 30–91) in conjunction with AUD history data collected on the sample since 1990, we investigated associations of COVID-19 related stressors and coping activities with changes in drunkenness frequency since the start of the pandemic. Analyses were conducted for those without a history of AUD (*N*: 645) and three groups of participants with a history of AUD prior to the start of the pandemic: (1) those experiencing AUD symptoms (*N*: 606), (2) those in remission who were drinking (*N*: 231), and (3) those in remission who were abstinent (had not consumed alcohol for 5+ years; *N*: 169). Gender-stratified models were also examined. Exploratory analyses examined the moderating effects of ‘problematic alcohol use’ polygenic risk scores (PRS) and neural connectivity (i.e., posterior interhemispheric alpha EEG coherence) on associations between COVID-19 stressors and coping activities with changes in the frequency of drunkenness. Increases in drunkenness frequency since the start of the pandemic were higher among those with a lifetime AUD diagnosis experiencing symptoms prior to the start of the pandemic (14% reported increased drunkenness) when compared to those without a history of AUD (5% reported increased drunkenness). Among individuals in remission from AUD prior to the start of the pandemic, rates of increased drunkenness were 10% for those who were drinking pre-pandemic and 4% for those who had previously been abstinent. Across all groups, women reported nominally greater increases in drunkenness frequency when compared with men, although only women experiencing pre-pandemic AUD symptoms reported significantly greater rates of increased drunkenness since the start of the pandemic compared to men in this group (17% of women vs. 5% of men). Among those without a prior history of AUD, associations between COVID-19 risk and protective factors with increases in drunkenness frequency were not observed. Among all groups with a history of AUD (including those with AUD symptoms and those remitted from AUD), *perceived stress* was associated with increases in drunkenness. Among the remitted-abstinent group, *essential worker status* was associated with increases in drunkenness. Gender differences in these associations were observed: among women in the remitted-abstinent group, *essential worker status, perceived stress, media consumption*, and *decreased social interactions* were associated with increases in drunkenness. Among men in the remitted-drinking group, *perceived stress* was associated with increases in drunkenness, and increased *relationship quality* was associated with decreases in drunkenness. Exploratory analyses indicated that associations between *family illness or death* with increases in drunkenness and increased *relationship quality* with decreases in drunkenness were more pronounced among the remitted-drinking participants with higher PRS. Associations between *family illness or death, media consumption*, and *economic hardships* with increases in drunkenness and *healthy coping* with decreases in drunkenness were more pronounced among the remitted-abstinent group with lower interhemispheric alpha EEG connectivity. Our results demonstrated that *only* individuals with pre-pandemic AUD symptoms reported greater increases in drunkenness frequency since the start of the COVID-19 pandemic compared to those without a lifetime history of AUD. This increase was more pronounced among women than men in this group. However, COVID-19-related stressors and coping activities were associated with changes in the frequency of drunkenness among all groups of participants with a prior history of AUD, including those experiencing AUD symptoms, as well as abstinent and non-abstinent participants in remission. *Perceived stress, essential worker status, media consumption*, *social connections* (especially for women), and *relationship quality* (especially for men) are specific areas of focus for designing intervention and prevention strategies aimed at reducing pandemic-related alcohol misuse among this particularly vulnerable group. Interestingly, these associations were not observed for individuals without a prior history of AUD, supporting prior literature that demonstrates that widespread stressors (e.g., pandemics, terrorist attacks) disproportionately impact the mental health and alcohol use of those with a prior history of problems.

## Introduction

The COVID-19 pandemic has led to disruptions in daily social activities, schooling, and employment for millions of people globally. For some, the pandemic has also led to financial insecurity, exposure to potentially hazardous working conditions, illness, and the illness and/or loss of a loved one. Research has linked traumatic stress from previous viral outbreaks and other mass-traumatic events (e.g., SARS epidemic, 9/11 terrorist attacks, mass shootings, natural disasters) to increases in alcohol use [1–4], particularly in vulnerable groups such as those with a history of alcohol use disorders (AUD) [5]. Much less is known, however, about the risk and protective factors for alcohol use during and after prolonged stressors, such as the COVID-19 pandemic. While there is mixed evidence across different study populations [6, 7], several initial reports have suggested that rates of alcohol use have increased since the start of the COVID-19 pandemic for some vulnerable groups [8–15]. Researchers have posited a variety of COVID-19-related hardships, such as social disconnection, lack of access to healthcare services, and economic difficulties, as potential explanations for these increases. However, it remains unknown which specific types of stressful COVID-19-related experiences are associated with increases in alcohol misuse and what healthy coping strategies might mitigate risk.

Prior research has demonstrated that some groups are more vulnerable to stress-related increases in alcohol misuse. For example, stressful events are associated with the recurrence of AUD [16], which underscores concern about the COVID-19 pandemic’s effect on alcohol use among individuals with a history of AUD. One recent study found that approximately half of adults in recovery from a substance use disorder reported cravings during a pandemic isolation period and that their craving was prompted by loneliness, lack of support, and financial stress, among other factors [17]. In addition, during the COVID-19 pandemic, access to mutual-help groups and specialized AUD treatments may have diminished [18]. This has played out in some early data on individuals with a lifetime history of AUD, which showed that ~20% of participants reported increases in their alcohol use since the pandemic began, with a significant portion of individuals reporting decreased access to substance use disorder treatment. However, other data have shown that 88.9% of women and 88.8% of men in a national survey of adults with ‘resolved’ AUD (i.e., no longer meet criteria for AUD and who were not drinking heavily) reported that the COVID-19 pandemic had not affected their recovery at all, and that ‘return to drinking’ events were infrequent [19]. These mixed findings indicate that research is needed to understand the association between potential stressors related to the COVID-19 pandemic and increases in drinking among individuals with a past AUD, and differences between those in remission from AUD (including abstinent, low-risk, and high-risk drinkers, who are all considered “in remission” according to new NIAAA definitions [20]) and those who are experiencing symptoms of AUD.

Women may be particularly vulnerable to increases in drinking during the COVID-19 pandemic [21–23]. Two independent studies found that women were more likely than men to have a recurrence of AUD when experiencing interpersonal conflict, whereas men were more likely than women to have a recurrence of AUD in response to social isolation [21–23]. Higher trauma exposure was associated with a higher risk of relapse only in women [23]. Being married has been identified as a relapse risk factor for women but a protective factor for men [21]. Because gender differences are often not examined in studies of individuals with AUD, robust, nuanced, and consistent findings regarding the role of gender in the associations of stress with drinking outcomes are unavailable. Since the start of the COVID-19 pandemic, some studies have found greater increases in adverse drinking outcomes among women than men [9, 11]. Given the unique stressors experienced by women during the COVID-19 pandemic, primarily related to balancing work and caregiving duties, further research is needed regarding gender differences in the relationships between COVID-19 pandemic stressors and increases in drinking among individuals with past AUD.

In addition, individuals at heightened risk for AUD are more vulnerable to stress-related alcohol use. For example, genetic and/or neural risk factors for AUD have been shown to increase the association between traumatic stress and alcohol use problems [24, 25]. There is also a growing literature suggesting that individuals exposed to traumatic stress differ in terms of temporally sensitive EEG-based measures of neural functional connectivity [26–32] (i.e., EEG alpha coherence and alpha hypoconnectivity in the Default Mode Network [22]), which are also associated with heightened risk for alcohol use disorder [28–31]. EEG coherence, the degree of synchrony in brain oscillatory activity between neural networks in two brain regions, is a heritable measure of neural functional connectivity that has been studied extensively and is correlated with various aspects of cognitive functioning and psychopathology [33–35]. While decades of research have focused on genetic and neurocognitive differences observed among those with alcohol use problems, few studies have examined interactions between measures of brain functioning and social environmental factors (e.g., traumatic stress) with respect to risk for alcohol use/misuse.

Research teasing apart specific types of stressful COVID-19-related experiences associated with problematic alcohol use (e.g., social disconnection, economic hardships), and detailing how they interact with individualized risk factors (e.g., history of AUD, gender, polygenic and neural risk factors), will allow us to better understand strategies that may buffer against the re-emergence, exacerbation, or new development of AUD that may occur as the COVID-19 pandemic continues to unfold. This study analyzed new data collected during the first months of the COVID-19 pandemic from longtime participants in the Collaborative Study on the Genetics of Alcoholism (COGA) study, in conjunction with their data on AUD and drinking collected in prior assessments. The primary aim of this study was to examine the associations between COVID-19-related stressors and coping activities with changes in drunkenness frequency since the start of the pandemic among men and women with and without a history of AUD. Using information from earlier (pre-pandemic) data collections, we further categorized individuals as having had no prior history of AUD pre-pandemic, having been symptomatic of AUD pre-pandemic, having been in remission from AUD but drinking pre-pandemic, or having been in remission from AUD and abstinent pre-pandemic. Gender differences in the associations of COVID-19-related stressors and coping activities with changes in drunkenness frequency were also examined. An exploratory aim was to examine the roles that polygenic risk for problematic alcohol use [36] and neural connectivity (i.e., posterior alpha EEG interhemispheric coherence) played as moderators of these associations.

## Methods

Details on the *Collaborative Study on the Genetics of Alcoholism (COGA)* data collection and procedures have been published previously [37, 38]. Briefly, since 1990 over 17,000 individuals from families densely affected with AUD and from community comparison families have participated in the COGA study. Participants were administered a comprehensive evaluation that included clinical assessments of substance use and psychiatric disorders using the Semi-Structured Assessment for the Genetics of Alcoholism (SSAGA) [39] research interview. In a subset of families, DNA was collected, and a brain functioning protocol was administered that included measures of EEG during resting state, such as measures of neural connectivity. The current COGA protocol began in 2019 when the project turned to a one-time follow-up assessment (SSAGA interviews, questionnaires, and a brain functioning battery) of previous COGA participants currently in two life stages: participants aged 50 or older (born 1973 or earlier), the majority of whom have a lifetime history of AUD, and participants aged 30–40 (born 1982–1993) from a longitudinal study of youth and young adult offspring from COGA families, approximately half of whom have a lifetime history of AUD. In these samples, over 95% had DNA and GWAS (Genome-Wide Association Study) data available, and over 75% completed at least one EEG assessment. Further details on these earlier studies have been published [37, 38].

### COGA’s COVID-19 analytic sample

In May 2020, shortly after the pandemic began and the data collection for the latest lifespan protocol had just begun, we added adapted versions of the CoRonavIruS Health Impact Survey (CRISIS) PhenX questionnaire [40] and the ABCD (Adolescent Brain Cognitive Development Study) COVID questionnaire [41] to the COGA assessment protocol to capture major COVID-19 related stressors, healthy coping activities, and changes in alcohol use/misuse since the onset of the pandemic in March 2020. While data collection is ongoing, as of July 22, 2022, questionnaire data had been obtained on 1654 participants. Genome-wide association data are available in 96.1% of this sample, and EEG assessments are available in 75.7% of the sample. Of those who completed the COVID-19 questionnaire, 61.8% were female, 79% were White, and the average age was 50.7 (Table 1).

**Table 1:** Descriptive characteristics of COGA’s lifespan project COVID-19 sample, stratified by pre-pandemic AUD status.

	All participants (*N* = 1651)	No past AUD (*N*: 645)	Current AUD symptoms (*N*: 606)	Remitted AUD, Drinking (*N*: 231)	Remitted AUD, Not-Drinking (*N*: 169)
**Socio-demographics**					
* Female (%)*	61.8	74.9^a^	51.7	60.2	50.3
* Self-reported Black (%)*	16.1	21.2^a^	15.8	8.2	7.7
* Self-reported White (%)*	79.0	74.7	77.6	88.3	88.8
* Self-reported Hispanic (%)*	7.9	6.0	10.1	7.8	6.5
* Age (Mean, SD)*	50.7 (15.8)	49.7 (16.6)	49.3 (14.5)	55.7 (13.4)	63.8 (11.8)^a^
**AUD Remission Status**					
* No past AUD (%)*	39.2	100.0^a^	0	0	0
* Current AUD symptoms (%)*	36.6	0	100.0^a^	0	0
* Remitted AUD, drinking (%)*	14.0	0	0	100.0^a^	0
* Remitted AUD, nondrinking (%)*	10.2	0	0	0	100.0^a^
**Alcohol related history (lifetime)**					
* Age of onset of AUD (Mean, SD)*	22.2 (6.4)	---	22.0 (6.2)	21.1 (4.7)	23.4 (7.6)
* Maximum number of DSM-5 AUD symptoms (0-11)*	3.5 (3.5)	0.4 (0.6)^a^	5.3 (2.9)	4.3 (2.6)	8.1 (2.9)
* Maximum drinks consumed in a 24-h period (Mean, SD)*	17.8 (15.2)	8.8 (8.6)^a^	23.3 (15.1)	19.4 (13.8)	29.8 (18.5)
* Age at first whole drink (Mean, SD)*	16.3 (2.6)	17.2 (2.6)^a^	15.4 (2.3)	15.9 (2.6)	15.7 (1.9)
* Age at last drink (Mean, SD)*	34.3 (8.9)	33.9 (9.9)	33.5 (7.8)	37.2 (7.7)	29.8 (18.5)
* Maximum number of AUD symptoms (Mean, SD)*	2.3 (2.2)	0.4 (0.6)	3.5 (1.8)	2.8 (1.7)	5.2 (1.9)
**Substance use history (lifetime)**					
* Ever smoked cigarettes (%)*	82.3	65.3^a^	94.2	90.8	96.9
* Ever used cannabis (%)*	67.0	54.3^a^	91.6	83.5	87.0
* Ever use cocaine (%)*	31.3	10.7^a^	54.6	43.3	63.3
* Ever used opiates (%)*	18.4	4.3^a^	36.3	20.8	43.2

^a^Significantly different *p* < 0.001 from other groups.

### History of DSM-5 AUD symptoms and remission ascertained from the SSAGA interviews prior to the COVID-19 pandemic

From these data, participants were designated as having (0) no lifetime history of AUD (*N*: 645) or having had a lifetime history of AUD (*N*: 1006), which included (1) those experiencing AUD symptoms at the time of COVID-19 assessments (*N*: 602–606, *see note below*), (2) those in remission (who no longer have symptoms of AUD other than craving) but are drinking; *N*: 231), (3) those in remission who have not consumed alcohol at all for 5+ years (*N*: 169). Four participants were in remission (i.e., no AUD symptoms except craving) and had not consumed alcohol at all for <1 year. ***Note***, given this <1 year duration of remission, and the small sample size, these individuals were included in the category along with participants currently experiencing symptoms. In light of interest in the alcohol research field of non-abstinent remission from AUD [20], we retained ‘remitted drinkers’ and ‘remitted non-drinkers’ as separate groups for analysis despite the relatively small sample size. These data were missing for three participants, who were excluded from the current study’s analyses.

***COGA’s COVID-19 questionnaires*** included 33 COVID-19-related stress and coping activities items. Assessment items covered perceived stresses, social disconnection, relationship quality, COVID-19 illness in the respondents and their family members (including prolonged hospitalization or death due to COVID-19 illness), economic hardships resulting from the pandemic (e.g., job loss, loss of household income, food insecurity), and coping strategies as shown in Supplementary Table 1. In our full analytic sample, the reliability of the items included in the COVID-19 questionnaires was good (Cronbach’s alpha: 0.82). To capture the shared variance represented by these items and to reduce the multiple test burden (i.e., reduce the number of independent variables and accordingly tests of association included in statistical models), each item in Supplementary Table 1 was entered into an exploratory factor analysis. Items were recoded such that higher values represented an endorsement of each stressor or coping activity. Change in frequency of drunkenness since the beginning of the COVID-19 pandemic was determined using a retrospective question asking if/how current alcohol use differed from the participant’s typical frequency of drunkenness prior to March 1, 2020 (“Compared to your [frequency of drunkenness] before the pandemic began in March 2020, was this [the same, less, more]?”). Self-reported changes in drunkenness were coded with possible values ranging from less than pre-pandemic use (-1), no change in use (0), more than pre-pandemic use (1).

***Genotyping*** was performed using the Illumina 2.5M array, Illumina OmniExpress, Illumina 1M array, or the Affymetrix Smokescreen array; quality control and imputation have been previously described [42]. SNPs with a genotyping rate <98% or that violated Hardy-Weinberg equilibrium (*p* < 10^−^^6^), or with minor allele frequency (MAF) less than 3% were excluded from analyses. Mendelian inconsistencies were removed, after which data were imputed to 1000 genomes (EUR and AFR, Phase 3, b37, October 2014; build hg19) using SHAPEIT [43] and IMPUTE2 [44]. Following imputation, genotype probabilities ≥0.90 were changed to genotypes. Mendelian errors in the imputed SNPs were reviewed and resolved as described previously [42]. SNPs with an imputation information score <0.30 or MAF < 0.03 were excluded from subsequent analysis. Based on 1000genomesv3, principal components (PCs) derived from GWAS data were used to ‘assign’ ancestry in the genotyped sample. We generated polygenic risk scores (PRS) for problematic alcohol use from the largest available GWAS meta-analysis (with COGA data removed) [45]. We constructed PRS using PRS-CSx, which applies a Bayesian regression with a continuous shrinkage parameter to GWAS summary statistics from multiple populations simultaneously [46, 47]. PRS were computed separately for each group of European ancestry and African ancestry participants.

***EEG recording and processing*** have been detailed previously [48]. Briefly, resting (eyes-closed) EEG was recorded for 4.25 min; a continuous interval of 256 s was analyzed. Conventional Fourier transform methods [49] were used to calculate coherence, and bipolar electrode pairs were derived to reduce volume conduction effects. Posterior interhemispheric (P4-O2--P3-O1) alpha band (7–12 Hz) coherence was used in the current analyses based on prior studies demonstrating the relationship with AUD [28–31]. Posterior alpha coherence was measured at least once since the start of COGA data collection on 75.7% of the analytic subsample. For individuals who had more than one EEG assessment, their most recent assessment was used. Among the analytic sample (*N*: 1250), those with EEG data and those without EEG data were similar in terms of frequency of women, Black and Hispanic participants, but the EEG subsample was more likely to be younger (*p* < 0.001).

### Statistical analysis

Structural equation models in Mplus *v8.5* were employed to examine the main effects of nine COVID-19-related stress and healthy coping latent factors (initially estimated in independent models) on change in drunkenness frequency. Stratified analyses were conducted for those without a prior history of AUD (0) and three groups of participants with a lifetime history of AUD, including (1) those who were experiencing AUD symptoms pre-pandemic, (2) those who were in remission from AUD and drinking, and (3) those in remission and had not consumed alcohol at all for 1+ years. Further, gender-stratified models were examined. Exploratory models examined multiplicative interactions between COVID-19-related stress and coping latent factors with problematic alcohol use PRS (z-score standardized) and low alpha EEG interhemispheric coherence (z-score standardized).

All models were adjusted for gender (in non-stratified models), self-reported identification as Black and/or Hispanic, study site, age, age cohort (0: born from 1982 to 1993, 1: born 1973 or earlier), and in genomic models, ancestral PCs1-3. For EEG coherence analyses, age at EEG assessment was also included as a covariate. All cross-term interactions (PRS*gender, COVID-19 latent factors*gender, COVID-19 latent factors*age, etc.) were included. All models were adjusted for familial clustering. Models were run simultaneously (i.e., all intercorrelations among factors accounted for), thus estimates are adjusted for all parameters and standardized. Given multiple comparisons across models, a Bonferroni correction adjusting for nine correlated latent factors across four groups was applied (*p* < *0.001*). While primary and stratified models had acceptable statistical power (alpha > 0.80), statistical power was weaker for the interaction models, including PRS and EEG variables. This was the primary reason we included these as ‘exploratory models’ that will require replication in larger samples.

## Results

Descriptive characteristics of the analytic sample are displayed in Table 1. Briefly, the average age of participants was 51; 62% were women, 16% self-identified as Black, and 8% self-identified as Hispanic (not mutually exclusive categories). Differences in characteristics of each analytic group as a function of past AUD history are depicted in Table 1. Individuals without a history of AUD were more likely to be women and Black and had less severe alcohol-related history and other substance use, compared to individuals with a history of AUD (symptomatic or in remission) and were younger compared only to abstinent individuals in remission from AUD.

**Fig. 1:**
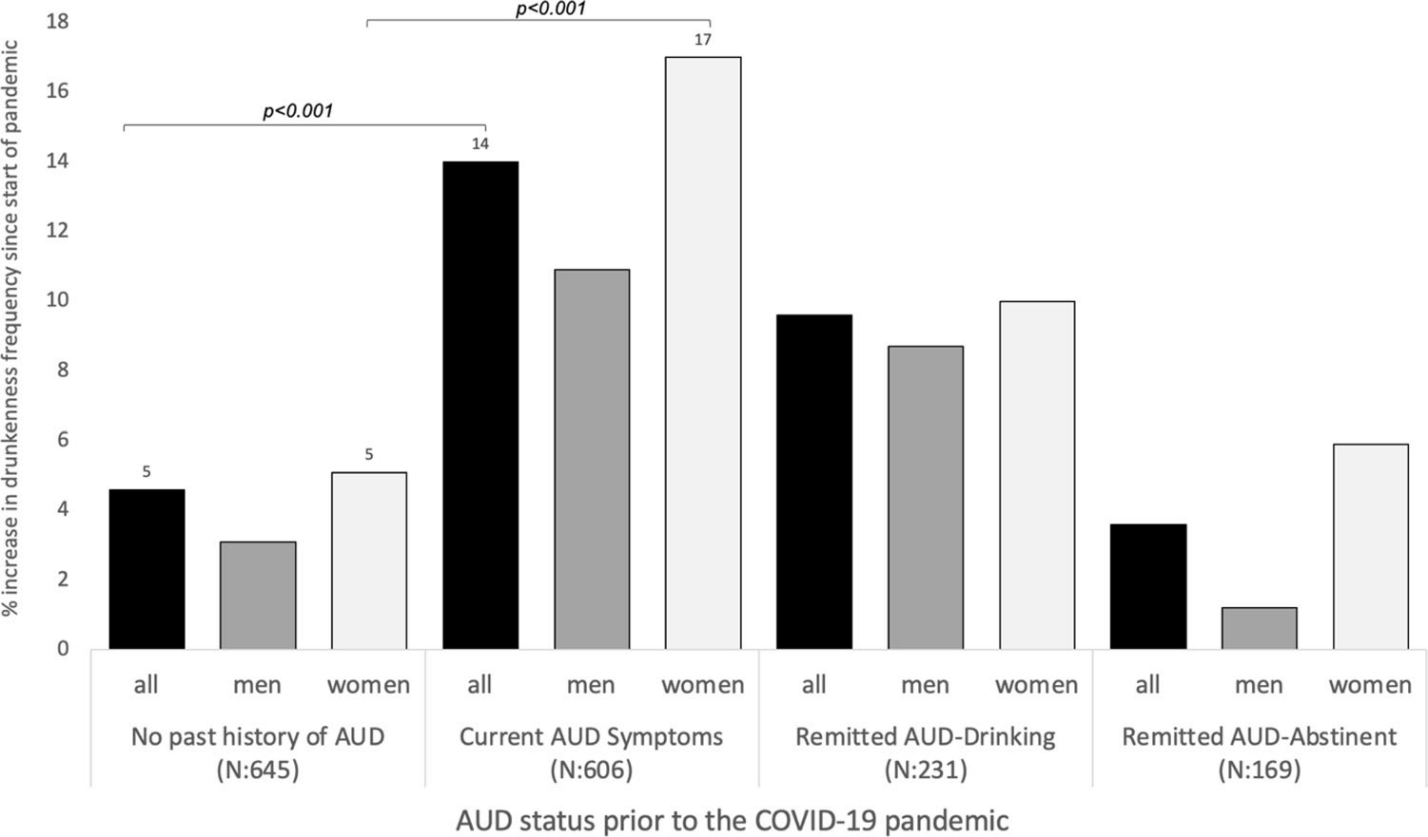
Reported increases in drunkenness frequency by AUD status and sex. AUD alcohol use disorder. No past history of AUD: no lifetime history. Current AUD Symptoms: have AUD symptoms at the time of COVID-19 assessments. Remitted AUD-Drinking: in remission with no AUD symptoms besides craving but are drinking. Remitted AUD-Abstinent: in remission and have not consumed alcohol for 5+ years.

Increases in drunkenness frequency since the start of the pandemic as a function of pre-pandemic AUD status are displayed in Fig. 1. Increases in drunkenness frequency since the start of the pandemic were significantly greater (*p* < 0.001) among those experiencing AUD symptoms prior to the start of the pandemic (14% report increased frequency of drunkenness) when compared to those without a history of AUD (5% report increased frequency drunkenness). Among individuals in remission from AUD prior to the start of the pandemic, rates of increased drunkenness were 10% for those who were drinking pre-pandemic and 4% for those who had previously been abstinent. Across all groups, women reported nominally greater increases in drunkenness frequency when compared with men; however, only women experiencing pre-pandemic AUD symptoms reported significantly greater (*p* < 0.001) rates of increased drunkenness since the start of the pandemic compared to women without a history of AUD (17% vs. 5%).

To capture the shared variance represented by the 33 COVID-19-related stress and coping activities items shown in Supplementary Table 1 and to reduce the multiple test burden, each item was entered into an exploratory factor analysis. While models ranging from 7 to 11 factors all provided a good fit to the data (Supplementary Tables 4–6), a 9-factor solution provided the best balance of model fit and interpretability. To obtain factor scores, we subsequently conducted a confirmatory factor analysis (CFA) including 9 latent factors indexing COVID-19 related (1) illness and severity, (2) family member illness and death, (3) media consumption, (4) perceived stress, (5) economic hardship, (6) healthy coping activities, (7) relationship quality, (8) social disconnection, and (9) essential worker status. Several of the COVID-19-related factors were correlated (Supplementary Table 2); among the most highly correlated factors were *perceived stress* with *social disconnection* (0.51), *media consumption* (0.34), and *economic hardships* (0.23). *Essential worker status* was also highly correlated with *COVID illness* (0.45).

**Fig. 2:**
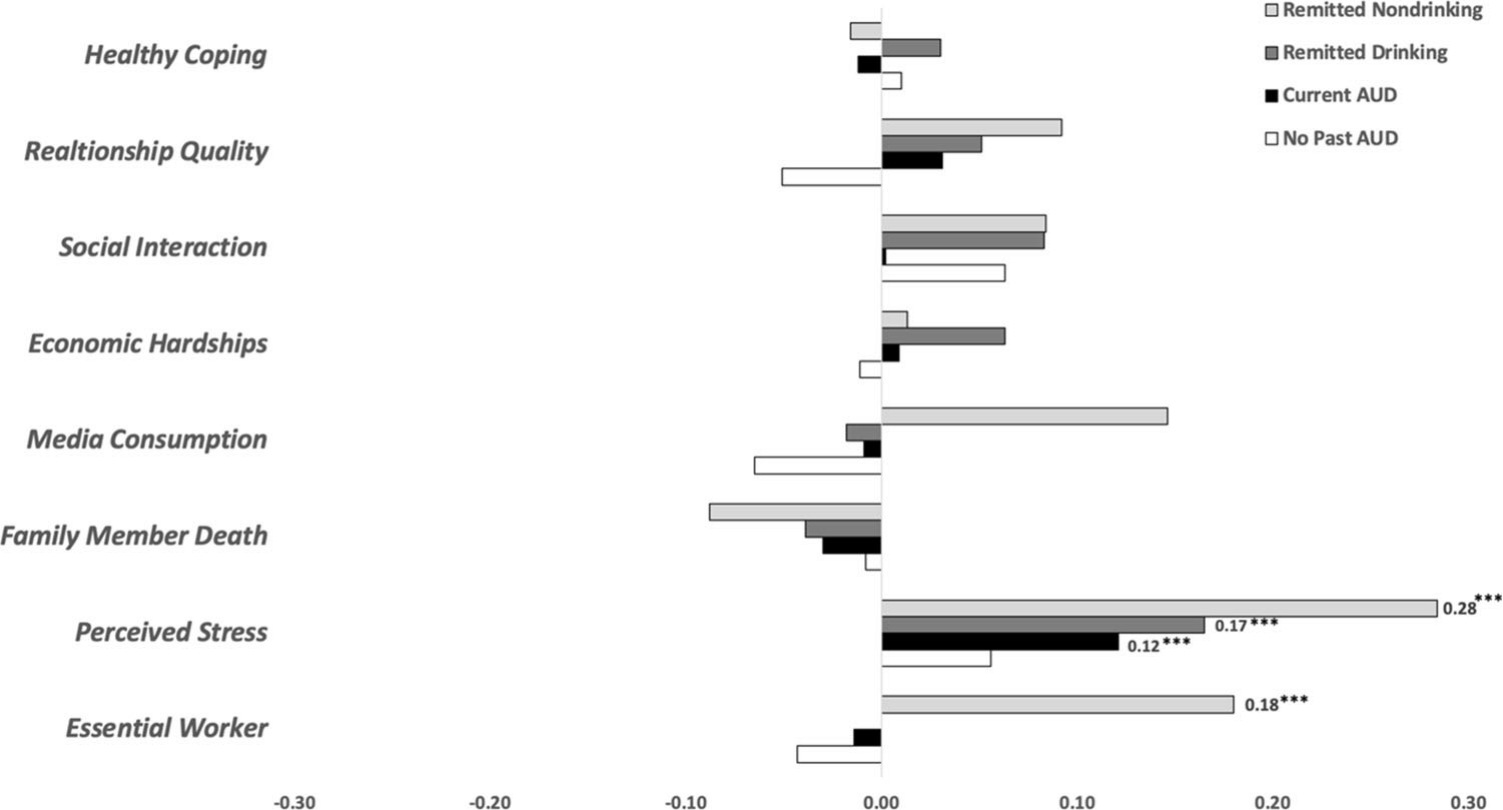
Risk and protective factors associated with reported changes in drunkenness frequency since March 2020 among COGA participants by pre-pandemic AUD status. The primary outcome variable used in the analytic models was determined using a retrospective question asking if/how current alcohol use differed from the participant’s typical frequency of drunkenness prior to March 1, 2020 (“Compared to your [frequency of drunkenness] before the pandemic began in March 2020, was this: [the same, less, more]?”). This was recomputed to represent whether the participant’s frequency of drunkenness was reduced from pre-pandemic levels (−1), the same as pre-pandemic levels (0), or increased from pre-pandemic levels (1). This figure depicts group-level “change” in terms of increased frequency of drunkenness since the beginning of the pandemic (beta coefficients ranging from 0 to 0.30, right-hand side of the figure) or decreased frequency of drunkenness since the beginning of the pandemic (beta coefficients ranging from 0 to −0.30, left-hand side of the figure).

The main effects of COVID-19-related stress and healthy coping latent factors on change in drunkenness frequency are displayed in Table 2 and Fig. 2. Note, Fig. 2 depicts group-level change in terms of participants who reported that their frequency of drunkenness has *increased* or *decreased* since the start of the pandemic. Among those without a prior history of AUD, associations between COVID-19 risk and protective factors with increases in drunkenness frequency were not observed. Among all groups with a history of AUD (including those with pre-pandemic AUD symptoms and those remitted from AUD), *perceived stress* was associated with increases in drunkenness. Among the remitted-abstinent group, *essential worker status* was associated with increases in drunkenness.

**Table 2:** Main effects of COVID-19 stress and coping factor scores on self-reported change in drunkenness frequency since the start of the pandemic among COGA participants without and with a history of AUD, including individuals with current symptoms of AUD and in abstinent and non-abstinent remission from AUD before the start of the COVID-19 pandemic.

	No past AUD (*N* = 645)		Current AUD (*N* = 606)		Remitted drinking (*N* = 231)		Remitted nondrinking (*N* = 169)	
All	*beta*	*p-value*	*beta*	*p-value*	*beta*	*p-value*	*beta*	*p-value*
** Essential worker**	−0.04	0.032	−0.01	0.639	0.00	0.997	**0.18**	**0.012**
** Perceived stress**	0.06	0.033	**0.12**	**<0.001**	**0.17**	**<0.001**	**0.28**	**<0.0001**
Family member death	−0.01	0.653	−0.03	0.324	−0.04	0.351	−0.09	0.315
Media consumption	−0.07	0.003	−0.01	0.758	−0.02	0.664	0.15	0.046
Economic hardships	−0.01	0.610	0.01	0.777	0.06	0.217	0.01	0.802
Social disconnection	0.06	0.038	0.00	0.960	0.08	0.212	0.08	0.404
Relationship quality	−0.05	0.054	−0.03	0.395	−0.05	0.291	−0.09	0.187
Healthy coping	0.01	0.658	−0.01	0.725	0.03	0.494	−0.02	0.826
**Female**	*beta*	*p-value*	*beta*	*p-value*	*beta*	*p-value*	*beta*	*p-value*
** Essential worker**	−0.05	0.051	−0.03	0.466	0.01	0.830	**0.22**	**<0.001**
** Perceived stress**	0.07	0.016	0.11	0.046	0.11	0.132	**0.45**	**<0.0001**
Family member death	−0.02	0.446	0.00	0.912	−0.06	0.301	−0.12	0.248
** Media consumption**	−0.07	0.011	−0.02	0.623	0.05	0.405	**0.23**	**<0.001**
Economic hardships	0.01	0.850	0.03	0.560	0.07	0.263	0.14	0.015
** Social disconnection**	0.04	0.304	−0.06	0.362	0.14	0.106	**0.22**	**<0.01**
Relationship quality	−0.04	0.255	0.03	0.502	−0.09	0.158	−0.01	0.882
Healthy coping	−0.01	0.839	−0.05	0.390	0.05	0.377	0.02	0.782
**Male**	*beta*	*p-value*	*beta*	*p-value*	*beta*	*p-value*	*beta*	*p-value*
Essential worker	−0.05	0.108	0.00	0.957	0.01	0.881	−0.17	0.002
** Perceived stress**	−0.04	0.419	0.12	0.018	**0.28**	**<0.0001**	−0.11	0.025
Family member death	0.01	0.722	−0.08	0.088	−0.03	0.584	−0.19	0.015
** Media consumption**	−0.07	0.058	0.02	0.638	−0.11	0.055	**0.20**	**<0.0001**
Economic hardships	−0.05	0.108	−0.01	0.811	0.08	0.350	−0.08	0.021
Social disconnection	0.09	0.043	0.05	0.381	0.01	0.910	0.06	0.594
** Relationship quality**	−0.09	0.015	−0.04	0.504	**−0.29**	**<0.0001**	−0.07	0.315
Healthy coping	0.05	0.121	0.04	0.391	0.06	0.370	0.12	0.013

All beta estimates are standardized (STDY); All models included the following covariates: sex, self-identification as Black, self-identification as Hispanic, life-stage (0: ages 30–40; 1: ages 50+), ancestral PC 1-3, age of EEG assessment. Bolded estimates withstood Bonferroni correction (*p* < 0.01).

**Fig. 3:**
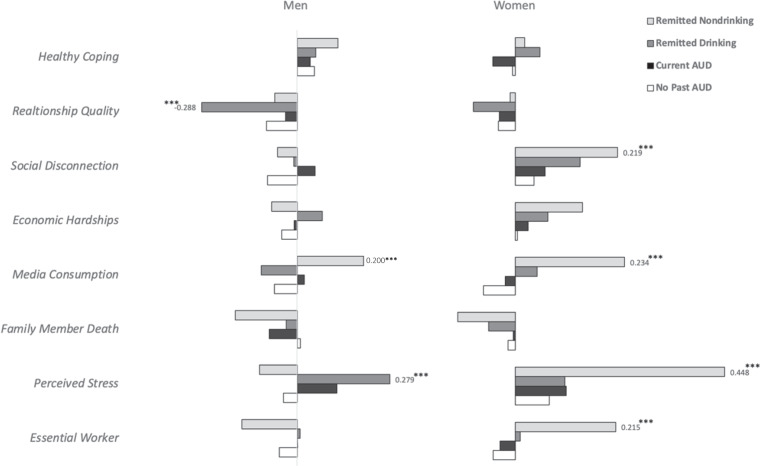
Risk and protective factors associated with reported increases in drunkenness frequency since March 2020 among COGA men and women, by pre-pandemic AUD status. The primary outcome variable used in the analytic models was determined using a retrospective question asking if/how current alcohol use differed from the participant’s typical frequency of drunkenness prior to March 1, 2020 (“Compared to your [frequency of drunkenness] before the pandemic began in March 2020, was this: [the same, less, more]?”). This was recomputed to represent whether the participant’s frequency of drunkenness was reduced from pre-pandemic levels (−1), the same as pre-pandemic levels (0), or increased from pre-pandemic levels (1). This figure depicts group-level “change” in terms of increased frequency of drunkenness since the beginning of the pandemic (beta coefficients ranging from 0 to 0.50, right-hand side of the figure) or decreased frequency of drunkenness since the beginning of the pandemic (beta coefficients ranging from 0 to −0.30, left-hand side of the figure).

Gender differences in these associations were observed (Table 2, Fig. 3). Among women in the remitted-abstinent group, *essential worker status, perceived stress, media consumption*, and *social disconnection* were associated with increases in drunkenness. Among men in the remitted-drinking group, *perceived stress* was associated with increases in drunkenness, and increased *relationship quality* was associated with decreases in drunkenness.

Exploratory analyses examining whether polygenic risk for ‘problematic alcohol use’ and/or low alpha EEG interhemispheric coherence moderate the associations of the COVID-19 factors and changes in drunkenness are presented in Supplementary Table 3. Exploratory analyses indicated that associations between *family illness or death* with increases in drunkenness and increased *relationship quality* with decreases in drunkenness were more pronounced among the remitted-drinking participants with higher PRS. Associations between *family illness or death, media consumption*, and *economic hardships* with increases in drunkenness and *healthy coping* with decreases in drunkenness were more pronounced among the remitted-abstinent group with lower interhemispheric alpha EEG connectivity. No main effects of PRS or neural connectivity on COVID-19 drinking outcomes were observed.

## Discussion

In this study, we examined the association of COVID-19-related stress and healthy coping activities with changes in drunkenness frequency since the start of the COVID-19 pandemic among participants with and without a history of AUD, including those experiencing symptoms and those in remission from AUD prior to the pandemic. Our results demonstrated that compared to those without a lifetime history of AUD, non-remitted individuals with a history of AUD reported greater increases in drunkenness frequency since the start of the COVID-19 pandemic; however, this difference was not observed among those who had been in remission from their AUD, regardless of their drinking status. COVID-19-related stressors and coping activities *were* associated with changes in the frequency of drunkenness among participants with symptoms of AUD and in both abstinent and non-abstinent participants in remission prior to the pandemic, but not among individuals without a past history of AUD. Among all groups with a history of AUD (including those with AUD symptoms and those remitted from AUD at the start of the pandemic), *perceived stress* was associated with increases in drunkenness. Among the remitted-abstinent group, *essential worker status* was associated with increases in drunkenness. These findings suggest that these may be specific areas of focus for designing intervention and prevention strategies aimed at reducing pandemic-related alcohol misuse among this particularly vulnerable group. Further, these findings support prior literature that demonstrates that widespread stressors (e.g., pandemics, terrorist attacks) disproportionately impact the alcohol use and mental health of those with a prior history of problems.

Significant main effects of COVID-19-related stressors and coping activities on changes in drunkenness were not observed among those without a history of AUD but *were* among those with a history of AUD, indicating that they may be more vulnerable to pandemic stress-related alcohol misuse. Specifically, among those with past AUD, *perceived stress* was associated with increases in drunkenness. Among the remitted-abstinent group, *essential worker status* was associated with increases in drunkenness. There are many potential explanations for why individuals with a history of AUD may be at increased risk for alcohol use following the stressful period that has encompassed the COVID-19 pandemic [7, 50–52], including facing a variety of stressful experiences related to the pandemic itself (i.e., serving as a frontline healthcare worker), sensitization by prior traumatic or stressful events (i.e., stress sensitization), genetic, and/or neural vulnerabilities. Previous work in COGA and other studies has also shown that many forms of stress (i.e., traumatic life events, SARS epidemic, 9/11 terrorist attacks) are associated with risks for alcohol misuse and problems [53–56], particularly among vulnerable individuals. Some studies examining mechanisms involved in increased stress and recurrence of AUD found an increased likelihood of cravings the evening following a stressful event and an increased likelihood of drinking the day after. Interestingly, these studies found that there is a protective effect for the length of time in recovery. Importantly, previous work has also shown that dimensions of interpersonal and social connections (i.e., romantic relationships, parent-child relationships, peer relationships, social support from religious communities) are associated with protection from alcohol misuse and problems [53–56]. Our findings also demonstrated that, among remitted-abstinent men, *relationship quality with family and friends* was related to decreases in drunkenness. Further, *social disconnection* was related to increases in drunkenness among remitted-abstinent women. Other studies have found that while general social support does not appear to be a protective factor against recurrence of AUD, positive familial and close relationship support does help to maintain good long-term AUD outcomes [57]. We note above that increases in the frequency of drunkenness were not observed for those without a history of AUD. Social events and celebrations have historically provided one setting in which individuals consume alcohol [58]. Given the pandemic’s interruption of these activities, this may provide one possible explanation for why those without a history of AUD did not increase their alcohol consumption during the pandemic, despite reporting stressful COVID-19-related experiences.

Gender differences were observed in both the change in frequency of drunkenness during the pandemic and the specific risk and protective factors associated with these increases. In the present study, women with AUD symptoms were more likely to report increases in drunkenness frequency since the start of the pandemic compared to women without a history of AUD. This was not observed for men. Interestingly, previous studies show that while there is an association between stress and increased alcohol consumption in both men and women, the same stressful life events may impact them differently [59, 60], with relapse to AUD more commonly reported in women than in men [33, 34, 61–64]. In addition to the increased drunkenness frequency observed among women with current AUD, the present study also found *essential worker status, perceived stress*, and *media consumption* were associated with increases in drunkenness among (remitted-abstinent) women, whereas *perceived stress* was associated with increases in drunkenness among (remitted-drinking) men. Further, *social disconnection* was associated with increases in drunkenness among (remitted-abstinent) women, whereas *relationship quality* was associated with decreases in drunkenness in (remitted-drinking) men. Past research on individuals with a history of AUD outside the context of the pandemic has found that gender differences regarding risk for recurrence of AUD are related to differential exposure to stressors, interpersonal relationships, and social isolation [21–23]. While this past research generally suggests that women may be more vulnerable to social/relationship stress-related drinking, our findings are less straightforward. Our interpretation of the current study findings is that both men and women in remission from AUD were vulnerable to the social-relationship consequences of the pandemic. It seems that they were *most vulnerable* to different aspects of these social-relationship consequences; for men increased relationship quality (i.e., “How has the quality of the relationship between you and members of your family changed?” “How has the quality of your relationships with your friends changed?”) was associated with decreases in drunkenness, whereas for women, decreased social connections (i.e., living alone, significantly decreased access to non-family social support, contacts outside the home significantly decreased, severely disrupted school/work/extracurricular routines) were associated with increases in drunkenness.

Several lines of evidence from gene x environment interaction (GxE) studies have shown that genetic risk factors moderate the associations of traumatic exposures with alcohol use behaviors. The current study supports and extends this research in demonstrating that polygenic risk for problematic alcohol use amplifies the association of COVID-19-related stressors (family death or illness) and increases in drunkenness and protective factors (relationship quality) with decreases in drunkenness among remitted individuals of European ancestry. These findings may be limited given the current methodological challenges applying genetic findings resulting from discovery samples of largely European ancestry to diverse populations, such as COGA’s lifespan study. While research examining the influence of interactions between neural risk factors and traumatic stress on substance use behaviors is less common, our prior research in COGA has demonstrated that neurophysiological and neurocognitive risk factors also amplify the association of traumatic stress on alcohol use behaviors [35, 65]. The current study extends this work by suggesting that low interhemispheric alpha EEG coherence amplifies the association of COVID-19-related stress (family death or illness, media consumption, and economic hardships) with increases in drunkenness, and COVID-19-related protective factors (healthy coping strategies) with decreases in drunkenness since the start of the pandemic. However, we note that these exploratory interaction effects need replication in larger samples. Importantly, among COGA participants ages 50–90, assessments of coherence could have occurred as long as 30 years prior to COVID-19 questionnaire data, which is likely to have an impact on these findings.

Limitations. The limitations of this study include a reliance on self-reported changes in drunkenness frequency, which are particularly relevant given the documented relationship between alcohol misuse and memory impairments [66]. Further, there is potential for a lack of generalizability of our study’s findings, particularly with respect to age, given that the mean age of the analytic sample was ~51 years old, and individuals who had remitted from AUD were older (remitted AUD, drinking group ~56 years old; remitted AUD, not-drinking group ~64 years old) than individuals with no past AUD (~50 years old) or with current symptoms of AUD (~49 years old). Future work should include more detailed subgroup analyses, including the identification of key stressors and healthy coping activities as a function of age and developmental stage, timing of lockdowns, seasonality, pandemic duration, and ethnicity. The CRISIS and ABCD questionnaires were initially selected to assess COVID-19-related stressors, coping and substance use. Among these questionnaire items, there were two items focused on alcohol use (changes in drinking frequency, changes in drunkenness frequency). We focused on changes in the frequency of drunkenness (vs. frequency of drinking) to get closer to the more ‘problematic’ use of alcohol. The data included in this manuscript is a ‘baseline’ assessment (May 2020–February 2021) of a longitudinal study designed to assess more nuanced aspects of alcohol use and misuse, including more comprehensive measures of drinking and drinking problems with a more precise description of time-periods to improve recall (e.g., AUDIT-P), assessed at three timepoints throughout the course of the pandemic. Thus, more detailed and objective measures of alcohol use and problems will be evaluated in future studies. In our full analytic sample, the reliability of the items included in the COVID-19 questionnaires was good (Cronbach’s alpha: 0.82). However, we will continue to monitor psychometric properties and conduct tests by AUD status, gender, age and ethnicity as the sample grows. Finally, in both the overall and gender-stratified analyses, change in drunkenness frequency among remitted-abstinent individuals was particularly affected by COVID risk and protective factors. We note that this group is significantly older and has a more severe alcohol-related history compared to other participants with a past AUD (Table 1). Both age and AUD severity likely have impacts on the associations of drunkenness with COVID risk and protective factors and should be explored in future studies.

In conclusion, this study has demonstrated that COVID-19-related stressors were associated with increased drunkenness frequency among COGA participants with a lifetime history of AUD, suggesting that they may be especially vulnerable to some stressors. Furthermore, this study revealed important gender differences in vulnerability to COVID-19-related stressors among those with a history of AUD. *Perceived stress, essential worker status, media consumption, social disconnection, and relationship quality* are specific areas of focus for designing intervention and prevention strategies aimed at reducing pandemic-related substance abuse.

### Supplementary information

Supplemental Tables

## References

[1] North CS, Pfefferbaum B (2013). Mental health response to community disasters: a systematic review. J Am Med Assoc.

[2] Neria Y, Nandi A, Galea S (2008). Post-traumatic stress disorder following disasters: a systematic review. Psychol Med..

[3] Bromet EJ, Atwoli L, Kawakami N, Navarro-Mateu F, Piotrowski P, King AJ (2017). Post-traumatic stress disorder associated with natural and human-made disasters in the World Mental Health Surveys. Psychol Med..

[4] Vlahov D, Galea S, Resnick H, Ahern J, Boscarino JA, Bucuvalas M (2002). Increased use of cigarettes, alcohol, and marijuana among Manhattan, New York, residents after the September 11th terrorist attacks. Am J Epidemiol.

[5] Meyers JL, Lowe SR, Eaton NR, Krueger R, Grant BF, Hasin D (2015). Childhood maltreatment, 9/11 exposure, and latent dimensions of psychopathology: a test of stress sensitization. J Psychiatr Res.

[6] Bade R, Simpson BS, Ghetia M, Nguyen L, White JM, Gerber C (2021). Changes in alcohol consumption associated with social distancing and self-isolation policies triggered by COVID-19 in South Australia: a wastewater analysis study. Addiction.

[7] Pabst A, Bollen Z, Creupelandt C, Fontesse S, Maurage P (2021). Alcohol consumption changes following COVID-19 lockdown among French-speaking Belgian individuals at risk for alcohol use disorder. Prog Neuropsychopharmacol Biol Psychiatry.

[8] Mallet J, Dubertret C, Le, Strat Y (2021). Addictions in the COVID-19 era: current evidence, future perspectives a comprehensive review. Prog Neuropsychopharmacol Biol Psychiatry..

[9] Barbosa C, Cowell A, Dowd W. How has drinking behavior changed during the COVID-19 pandemic? RTI International. 2020. https://www.rti.org/sites/default/files/covid19_alcohol_survey_webinar_slides_071420.pdf.

[10] Killgore WDS, Taylor EC, Cloonan SA, Dailey NS (2020). Psychological resilience during the COVID-19 lockdown. Psychiatry Res.

[11] Pollard MS, Tucker JS, Green HD (2020). Changes in adult alcohol use and consequences during the COVID-19 pandemic in the US. JAMA Netw Open.

[12] Oksanen A, Oksa R, Savela S, Mantere E, Savolainen I, Kaakinen M (2021). COVID-19 crisis and digital stressors at work: a longitudinal study on the Finnish working population. Comput Hum Behav..

[13] Van Nguyen H, Lan Nguyen H, Thi Minh Dao A, Van Nguyen T, The Nguyen P, Mai Le P (2022). The COVID-19 pandemic in Australia: public health responses, opportunities and challenges. Int J health Plan Manag.

[14] Mangot-Sala L, Tran KA, Smidt N, Liefbroer AC (2022). The impact of the COVID lockdown on alcohol consumption in the Netherlands. The role of living arrangements and social isolation. Drug Alcohol Depend.

[15] Sohi I, Chrystoja BR, Rehm J, Wells S, Monteiro M, Ali S (2022). Changes in alcohol use during the COVID- 19 pandemic and previous pandemics: a systematic review. Alcohol Clin Exp Res.

[16] Wemm SE, Larkin C, Hermes G, Tennen H, Sinha R (2019). A day-by-day prospective analysis of stress, craving and risk of next day alcohol intake during alcohol use disorder treatment. Drug Alcohol Depend.

[17] Bonny-Noach H, Gold D (2021). Addictive behaviors and craving during the COVID-19 pandemic of people who have recovered from substance use disorder. J Addict Dis.

[18] Rapoport R. “Every day is an emergency”: the pandemic is worsening psychiatric bed shortages nationwide. STAT news. 2021. https://www.statnews.com/2020/12/23/mental-health-covid19-psychiatric-beds/.

[19] Gilbert PA, Soweid L, Kersten SK, Brown G, Zemore SE, Mulia N (2021). Maintaining recovery from alcohol use disorder during the COVID-19 pandemic: the importance of recovery capital. Drug Alcohol Depend.

[20] Hagman BT, Falk D, Litten R, Koob GF (2022). Defining recovery from alcohol use disorder: development of an NIAAA research definition. Am J Psychiatry.

[21] Walitzer KS, Dearing RL (2006). Gender differences in alcohol and substance use relapse. Clin Psychol Rev.

[22] Zywiak WH, Stout RL, Trefry WB, Glasser I, Connors GJ, Maisto SA (2006). Alcohol relapse repetition, gender, and predictive validity. J Subst Abus Treat.

[23] Heffner JL, Blom TJ, Anthenelli RM (2011). Gender differences in trauma history and symptoms as predictors of relapse to alcohol and drug use. Am J Addict.

[24] Meyers JL, Dick DM (2010). Genetic and environmental risk factors for adolescent-onset substance use disorders. Child Adolesc Psychiatr Clin N Am..

[25] Salvatore JE, Cho SBin, Dick DM (2017). Genes, environments, and sex differences in alcohol research. J Stud Alcohol Drugs.

[26] Porjesz B, Rangaswamy M, Kamarajan C, Jones KA, Padmanabhapillai A, Begleiter H (2005). The utility of neurophysiological markers in the study of alcoholism. Clin Neurophysiol..

[27] Rangaswamy M, Porjesz B (2014). Understanding alcohol use disorders with neuroelectrophysiology. Handb Clin Neurol.

[28] Park SM, Lee JY, Kim YJ, Lee JY, Jung HY, Sohn BK (2017). Neural connectivity in Internet gaming disorder and alcohol use disorder: a resting-state EEG coherence study. Sci Rep.

[29] Mumtaz W, Vuong PL, Xia L, Malik AS, Rashid RBA (2017). An EEG-based machine learning method to screen alcohol use disorder. Cogn Neurodyn.

[30] Kamarajan C, Ardekani BA, Pandey AK, Chorlian DB, Kinreich S, et al. Random forest classification of alcohol use disorder using EEG source functional connectivity, neuropsychological functioning, and impulsivity measures. Behav Sci*.* 2020;10:62. 10.3390/bs10030062. 10.3390/bs10030062 PMC7139327 32121585

[31] Cardenas VA, Price M, Fein G (2018). EEG coherence related to fMRI resting state synchrony in long-term abstinent alcoholics. Neuroimage Clin.

[32] Clancy KJ, Andrzejewski JA, Simon J, Ding M, Schmidt NB, Li W (2020). Posttraumatic stress disorder is associated with a dysrhythmia across the visual cortex and the default mode network. eNeuro.

[33] Evans SM, Levin FR (2011). Response to alcohol in women: role of the menstrual cycle and a family history of alcoholism. Drug Alcohol Depend.

[34] Kiesner J (2012). Affective response to the menstrual cycle as a predictor of self-reported affective response to alcohol and alcohol use. Arch Women’s Ment Health.

[35] Meyers J, McCutcheon VV, Pandey AK, Kamarajan C, Subbie S, Chorlian D (2019). Early sexual trauma exposure and neural response inhibition in adolescence and young adults: trajectories of frontal theta oscillations during a go/no-go task. J Am Acad Child Adolesc Psychiatry.

[36] Barr PB, Ksinan A, Su J, Johnson EC, Meyers JL, Wetherill L (2020). Using polygenic scores for identifying individuals at increased risk of substance use disorders in clinical and population samples. Transl Psychiatry.

[37] Schuckit MA, Smith TL, Danko G, Kramer J, Bucholz KK, McCutcheon V (2018). A 22-year follow-up (range 16 to 23) of original subjects with baseline alcohol use disorders from the collaborative study on genetics of alcoholism. Alcohol Clin Exp Res.

[38] Bucholz KK, McCutcheon VV, Agrawal A, Dick DM, Hesselbrock VM, Kramer JR, et al. Comparison of parent, peer, psychiatric, and cannabis use influences across stages of offspring alcohol involvement: evidence from the COGA prospective study. Alcohol Clin Exp Res. 2017;41:359–368. 10.1111/acer.13293. 10.1111/acer.13293 PMC5272776 28073157

[39] Bucholz KK, Cadoret R, Cloninger CR, Dinwiddie SH, Hesselbrock VM, Nurnberger JI (1994). A new, semi-structured psychiatric interview for use in genetic linkage studies: a report on the reliability of the SSAGA. J Stud Alcohol.

[40] The CoRonavIruS Health Impact Survey (CRISIS) V0.3. https://www.phenxtoolkit.org/toolkit_content/PDF/CRISIS_Baseline_Adult.pdf.

[41] Pelham WE, Tapert SF, Gonzalez MR, McCabe CJ, Lisdahl KM, Alzueta E (2021). Early adolescent substance use before and during the COVID-19 pandemic: a longitudinal survey in the ABCD study cohort. J Adolesc Health.

[42] Lai D, Wetherill L, Bertelsen S, Carey CE, Kamarajan C, Kapoor M (2019). Genome-wide association studies of alcohol dependence, DSM-IV criterion count and individual criteria. Genes Brain Behav.

[43] Delaneau O, Howie B, Cox AJ, Zagury J-F, Marchini J (2013). Haplotype estimation using sequencing reads. Am J Hum Genet.

[44] Das S, Forer L, Schönherr S, Sidore C, Locke AE, Kwong A (2016). Next-generation genotype imputation service and methods. Nat Genet.

[45] Zhou H, Sealock JM, Sanchez-Roige S, Clarke TK, Levey DF, Cheng Z (2020). Genome-wide meta-analysis of problematic alcohol use in 435,563 individuals yields insights into biology and relationships with other traits. Nat Neurosci.

[46] Ge T, Chen C-Y, Ni Y, Feng Y-CA, Smoller JW (2019). Polygenic prediction via Bayesian regression and continuous shrinkage priors. Nat Commun.

[47] Ruan Y, Lin YF, Feng YA, Chen CY, Lam M, Guo Z (2022). Improving polygenic prediction in ancestrally diverse populations. Nat Genet.

[48] Chorlian DB, Rangaswamy M, Porjesz B (2009). EEG coherence: topography and frequency structure. Exp Brain Res.

[49] Nunez PL, Srinivasan R, Westdorp AF, Wijesinghe RS, Tucker DM, Silberstein RB (1997). EEG coherency. I: Statistics, reference electrode, volume conduction, Laplacians, cortical imaging, and interpretation at multiple scales. Electroencephalogr Clin Neurophysiol.

[50] Gavurova B, Ivankova V, Rigelsky M (2020). Relationships between perceived stress, depression and alcohol use disorders in university students during the COVID-19 pandemic: a socio-economic dimension. Int J Environ Res Public Health.

[51] Sallie SN, Ritou V, Bowden-Jones H, Voon V. Assessing international alcohol consumption patterns during isolation from the COVID-19 pandemic using an online survey: highlighting negative emotionality mechanisms. BMJ Open. 2020;10:e044276. 10.1136/bmjopen-2020-044276. 10.1136/bmjopen-2020-044276 PMC7692002 33243820

[52] Charles NE, Strong SJ, Burns LC, Bullerjahn MR, Serafine KM. Increased mood disorder symptoms, perceived stress, and alcohol use among college students during the COVID-19 pandemic. Psychiatry Res. 2021;296:113706. 10.1016/j.psychres.2021.113706. 10.1016/j.psychres.2021.113706 PMC7781902 33482422

[53] Meyers JL, Salvatore JE, Aliev F, Johnson EC, McCutcheon VV, Su J, et al. Psychosocial moderation of polygenic risk for cannabis involvement: the role of trauma exposure and frequency of religious service attendance. Transl Psychiatry. 2019;9:269. 10.1038/s41398-019-0598-z. 10.1038/s41398-019-0598-z PMC6803671 31636251

[54] Salvatore JE, Thomas NS, Cho SB, Adkins A, Kendler KS, Dick DM (2016). The role of romantic relationship status in pathways of risk for emerging adult alcohol use. Psychol Addict Behav.

[55] Salvatore JE, Kendler KS, Dick DM (2014). Romantic relationship status and alcohol use and problems across the first year of college. J Stud Alcohol Drugs.

[56] Johnson EC, Tillman R, Aliev F, Meyers JL, Salvatore JE, Anokhin AP (2019). Exploring the relationship between polygenic risk for cannabis use, peer cannabis use and the longitudinal course of cannabis involvement. Addiction.

[57] McCrady BS, Flanagan JC (2021). The role of the family in alcohol use disorder recovery for adults. Alcohol Res..

[58] Cooper ML, Kuntsche E, Levitt A, Barber LL, Wolf S. 'Motivational Models of Substance Use: A Review of Theory and Research on Motives for Using Alcohol, Marijuana, and Tobacco'. In: Kenneth JS, ed. The Oxford Handbook of Substance Use and Substance Use Disorders: Volume 1, Oxford Library of Psychology (2016; online edn, Oxford Academic, 1 July 2014), 10.1093/oxfordhb/9780199381678.013.017.

[59] Verplaetse TL, Cosgrove KP, Tanabe J, McKee SA (2021). Sex/gender differences in brain function and structure in alcohol use: a narrative review of neuroimaging findings over the last 10 years. J Neurosci Res.

[60] Koob GF, White A. Alcohol and the female brain. 2017 National Conference on Alcohol and Opioid Use in Women & Girls: advances in prevention, treatment, and recovery research. 2017. Washington DC, MA.

[61] Verplaetse TL, Moore KE, Pittman BP, Roberts W, Oberleitner LM, Smith PH (2018). Intersection of stress and gender in association with transitions in past year DSM-5 substance use disorder diagnoses in the United States. Chronic Stress.

[62] Becker JB, Koob GF (2016). Sex differences in animal models: focus on addiction. Pharm Rev.

[63] Mello NK, Mendelson JH, Lex BW (1990). Alcohol use and premenstrual symptoms in social drinkers. Psychopharmacol.

[64] Svikis DS, Miles DR, Haug NA, Perry B, Hoehn-Saric R, McLeod D (2006). Premenstrual symptomatology, alcohol consumption, and family history of alcoholism in women with premenstrual syndrome. J Stud Alcohol.

[65] Subbie-Saenz de Viteri S, Pandey A, Pandey G, Kamarajan C, Smith R, Anokhin A, et al. Pathways to post-traumatic stress disorder and alcohol dependence: trauma, executive functioning, and family history of alcoholism in adolescents and young adults. Brain Behav. 2020;10:e01789. 10.1002/brb3.1789. 10.1002/brb3.1789 PMC7667345 32990406

[66] Le Berre A-P, Fama R, Sullivan EV (2017). Executive functions, memory, and social cognitive deficits and recovery in chronic alcoholism: a critical review to inform future research. Alcohol Clin Exp Res.

