# Supplementary material for: COVID-19 pandemic stressors are associated with reported increases in frequency of drunkenness among individuals with a history of alcohol use disorder
Source: Transl Psychiatry. 2023 Oct 6;13:311. doi: 10.1038/s41398-023-02577-1 (PMC10558437; doi:10.1038/s41398-023-02577-1)
Supplement: Supplementary file 1 — Supplemental Tables [file 41398_2023_2577_MOESM1_ESM.docx]

| **Supplemental Table 1.** COGA COVID-19 pandemic stress and coping activity questionnaire items, stratified by pre-pandemic AUD status | | | | |
| --- | --- | --- | --- | --- |
|  | **No past history of AUD**  **(N: 645)** | **Current AUD sx**  **(N: 606)** | **Remitted AUD, Drinking**  **(N: 231)** | **Remitted AUD,**  **Abstinent (N:169)** |
| **COVID illness** | % | % | % | % |
| *COVID diagnosis or symptoms consistent with diagnosis* | 15.0 | 9.1 | 11.7 | 10.7 |
| *Severe COVID symptoms* | 4.3 | 3.8 | 3.4 | 3.0 |
| **Frontline worker status** |  |  |  |  |
| *Essential worker status* | 36.6 | 36.3 | 39.0 | 29.6 |
| *Healthcare worker status* | 9.5 | 7.6 | 8.2 | 5.9 |
| **Perceived stress** |  |  |  |  |
| *Experience of stress (severe)* | 4.8 | 9.4* | 6.5 | 6.5 |
| *COVID restrictions stressful (extreme)* | 3.6 | 5.1 | 3.9 | 5.3 |
| *Physical health worries (severe)* | 5.4 | 8.4 | 5.2 | 4.7 |
| *Mental health worries (severe)* | 4.2 | 8.4 | 4.8 | 5.9 |
| **Family member COVID illness** |  |  |  |  |
| *Family member symptomatic due to COVID* | 37.1 | 24.1 | 27.7 | 21.9 |
| *Family member hospitalized due to COVID* | 12.7 | 10.6 | 8.7 | 5.3 |
| *Family member died due to COVID* | 7.3 | 5.4 | 6.5 | 3.6 |
| **COVID related media consumption** |  |  |  |  |
| *Reading/Talking about COVID (all the time)* | 8.2 | 13.0 | 15.6 | 10.1 |
| *TV/Digital media/Social Media about COVID (all the time)* | 4.7 | 8.4 | 10.4 | 6.5 |
| **Economic hardships** | - |  |  |  |
| *Unemployed due to COVID* | 5.9 | 10.6* | 6.5 | 7.1 |
| *COVID impacted personal income (major loss of income)* | 9.6 | 13.5 | 6.5 | 10.7 |
| *COVID impacted household income (major loss of income)* | 9.9 | 14.7 | 6.9 | 12.4 |
| *Food insecurity* | 10.7 | 17.7* | 10.0 | 10.7 |
| **Access to healthcare** |  |  |  |  |
| *Decreased access to medical health care (severe)* | 1.6 | 4.8* | 2.2 | 1.8 |
| *Decreased access to mental health care (severe)* | 1.2 | 4.5* | 1.7 | 0.6 |
| **Social disconnection** |  |  |  |  |
| *Living alone* | 17.2 | 18.3 | 16.9 | 20.7 |
| *Decreased access to non-family social support (severe)* | 6.4 | 10.7 | 9.1 | 11.2 |
| *Contacts outside home decreased (significantly)* | 44.2 | 48.2 | 47.6 | 55.6 |
| *Disrupted school, work, extracurricular routines (severe)* | 20.9 | 27.4 | 26.4 | 20.7 |
| **Relationship quality** | | | | |
| *Relationship quality family impacted (a lot worse, a lot better)* | 2.3, 5.0 | 4.8*, 4.1 | 2.2, 6.5 | 3.6, 3.6 |
| *Relationship quality friends impacted (a lot worse, a lot better)* | 3.6, 2.0 | 5.9, 1.3 | 5.6, 2.6 | 4.7, 3.0 |
| **Healthy coping activities** |  |  |  |  |
| *Taking media breaks* | 67.0 | 66.2 | 64.9 | 66.9 |
| *Social distanced activities* | 24.7 | 22.4 | 22.9 | 11.8* |
| *Breathing, Stretching, Meditating* | 62.9 | 54.6 | 48.9 | 53.8 |
| *Exercising* | 67.8 | 59.4 | 58.4 | 48.5 |
| *Hobbies* | 54.6 | 49.5 | 48.9 | 43.8 |
| *Healthy eating and sleeping behaviors* | 69.3 | 61.4 | 60.2 | 63.9 |
| *Relaxation* | 72.1 | 67.3 | 67.1 | 71.6 |
| *Connecting online/phone* | 78.0 | 73.6 | 71.0 | 70.4 |

| **Supplemental Table 2.** Correlations among COVID-19 stress and healthy coping activities factor scores | | | | | | | | | |
| --- | --- | --- | --- | --- | --- | --- | --- | --- | --- |
| Factor Scores | (1) | (2) | (3) | (4) | (5) | (6) | (7) | (8) | (9) |
| *(1) COVID illness* | 1 |  |  |  |  |  |  |  |  |
| *(2) Family Illness/death* | **-0.234^**^** | 1 |  |  |  |  |  |  |  |
| *(3) Media Consumption* | -0.048 | 0.045 | 1 |  |  |  |  |  |  |
| *(4) Perceived Stress* | -0.088 | **0.121^**^** | **0.335^**^** | 1 |  |  |  |  |  |
| *(5) Economic Hardships* | **-0.124^*^** | 0.034 | 0.043 | **0.227^**^** | 1 |  |  |  |  |
| *(6) Healthy Coping* | -0.021 | 0.038 | **0.113^**^** | **0.168^**^** | 0.046 | 1 |  |  |  |
| *(7) Relationship Quality* | -0.081 | -0.015 | **-0.098^**^** | **-0.252^**^** | **-0.064^*^** | 0.041 | 1 |  |  |
| *(8) Social Disconnection* | -0.062 | **0.079^**^** | **0.248^**^** | **0.513^**^** | **0.055^*^** | **0.163^**^** | **-0.257^**^** | 1 |  |
| *(9) Essential Worker* | **0.449**** | 0.024 | **-0.058^*^** | **-0.071^**^** | **0.077^**^** | -0.017 | 0.046 | **-0.052^*^** | 1 |

**Supplemental Table 3.** Exploratory analyses examining the roles that polygenic risk for ‘problematic alcohol use’^16^, and neural connectivity (i.e., alpha EEG interhemispheric coherence) play as moderators of the associations between COVID-19 related stress and coping factors and frequency of drunkenness since the start of the pandemic among COGA participants without and with a history of AUD, including those with current symptoms and both in abstinent and non-abstinent remission prior to the start of the pandemic.

**
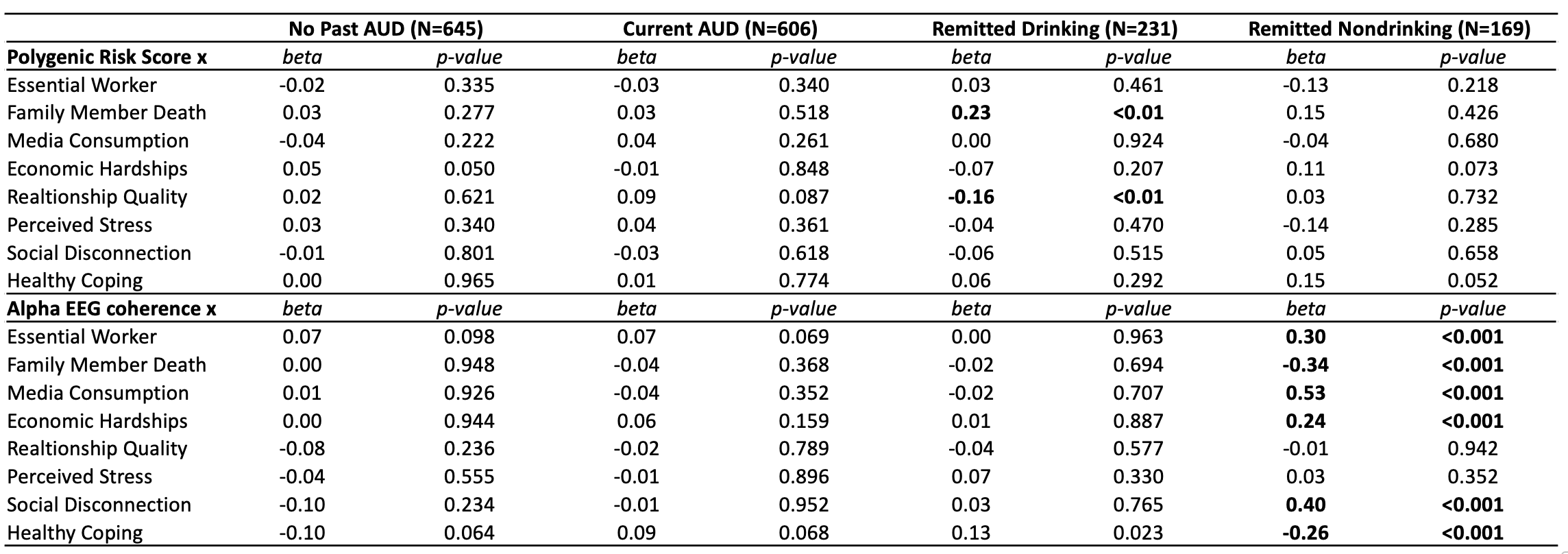
**

**Note:** All beta estimates are standardized (STDY); All models included the following covariates: sex, self-identification as Black, self-identification as Hispanic, life-stage (0: ages 30-40; 1: ages 50+), ancestral PC 1-3, age of EEG assessment. **Bolded** estimates withstood Bonferroni correction (p<0.001).

| **Supplemental Table 4.** Model Fit Statistics from Exploratory Factor Analysis | | | | |
| --- | --- | --- | --- | --- |
| *Factor Solutions* | *Parameters (#)* | *Chi-Square* | *Degrees of Freedom* | *P-value* |
| 1-factor | 99 | 4309.795 | 495 | <0.0001 |
| 2-factor | 131 | 3078.534 | 463 | <0.0001 |
| 3-factor | 162 | 1949.902 | 432 | <0.0001 |
| 4-factor | 192 | 1588.713 | 402 | <0.0001 |
| 5-factor | 221 | 1288.575 | 373 | <0.0001 |
| 6-factor | 249 | 1031.03 | 345 | <0.0001 |
| 7-factor | 276 | 803.964 | 318 | <0.0001 |
| 8-factor | 302 | 606.232 | 292 | <0.0001 |
| 9-factor | 327 | 488.941 | 267 | <0.0001 |
| 10-factor | 351 | 356.65 | 243 | <0.0001 |
| **11-factor** | **374** | **279.875** | **220** | **<0.010** |
| **12-factor** | **396** | **228.979** | **198** | **0.065** |
| **13-factor** | **417** | **193.565** | **177** | **0.187** |
| *Note: Models with 14 or greater factors did not converge* | | | | |

| **Supplemental Table 5.** Model Fit Statistics from Exploratory Factor Analysis | | | |
| --- | --- | --- | --- |
| *Models Compared* | *Chi-Square* | *Degrees of Freedom* | *P-Value* |
| 1-factor against 2-factor | 1231.261 | 32 | <0.0001 |
| 2-factor against 3-factor | 1128.632 | 31 | <0.0001 |
| 3-factor against 4-factor | 361.189 | 30 | <0.0001 |
| 4-factor against 5-factor | 300.138 | 29 | <0.0001 |
| 5-factor against 6-factor | 257.545 | 28 | <0.0001 |
| 6-factor against 7-factor | 227.066 | 27 | <0.0001 |
| 7-factor against 8-factor | 197.732 | 26 | <0.0001 |
| 8-factor against 9-factor | 117.29 | 25 | <0.0001 |
| 9-factor against 10-factor | 132.291 | 24 | <0.0001 |
| 10-factor against 11-factor | 76.776 | 23 | <0.0001 |
| 11-factor against 12-factor | 50.896 | 22 | <0.0010 |
| 12-factor against 13-factor | 35.414 | 21 | 0.0254 |

| **Supplemental Table 6.** Model Fit Statistics from Exploratory Factor Analysis | | | | | | | | |
| --- | --- | --- | --- | --- | --- | --- | --- | --- |
| *Factors* | *Par (#)* | *AIC* | *BIC* | *CFI* | *RMSEA* | *lower* | *upper* | *p-val* |
| 1-factor | 99 | 44130.309 | 44598.680 | 0.402 | 0.096 | 0.093 | 0.099 | <0.001 |
| 2-factor | 131 | 42963.048 | 43582.812 | 0.590 | 0.082 | 0.079 | 0.085 | <0.001 |
| 3-factor | 162 | 41896.416 | 42662.841 | 0.762 | 0.065 | 0.062 | 0.068 | <0.001 |
| 4-factor | 192 | 41595.228 | 42503.583 | 0.814 | 0.059 | 0.056 | 0.062 | <0.001 |
| 5-factor | 221 | 41353.090 | 42398.645 | 0.856 | 0.054 | 0.051 | 0.057 | 0.018 |
| 6-factor | 249 | 41151.545 | 42329.568 | 0.892 | 0.049 | 0.045 | 0.052 | 0.727 |
| 7-factor | 276 | 40978.479 | 42284.240 | 0.924 | 0.43 | 0.039 | 0.046 | 1.00 |
| 8-factor | 302 | 40832.746 | 42261.514 | 0.951 | 0.036 | 0.032 | 0.040 | 1.00 |
| **9-factor** | **327** | **40765.456** | **42312.499** | **0.965** | **0.031** | **0.027** | **0.036** | **1.00** |
| 10-factor | 351 | 40681.165 | 42341.752 | 0.982 | 0.024 | 0.018 | 0.029 | 1.00 |
| 11-factor | 374 | 40650.389 | 42419.790 | 0.991 | 0.018 | 0.011 | 0.024 | 1.00 |
| 12-factor | 396 | 40643.493 | 42516.976 | 0.995 | 0.014 | 0.000 | 0.021 | 1.00 |
| 13-factor | 417 | 40650.079 | 42622.914 | 0.997 | 0.011 | 0.000 | 0.019 | 1.00 |
